# Epigenetic modulation of cytokine expression in gastric cancer: influence on angiogenesis, metastasis and chemoresistance

**DOI:** 10.3389/fimmu.2024.1347530

**Published:** 2024-02-22

**Authors:** María Elena Reyes, Victoria Pulgar, Carolina Vivallo, Carmen Gloria Ili, Bárbara Mora-Lagos, Priscilla Brebi

**Affiliations:** ^1^ Instituto de Ciencias Biomédicas, Facultad de Ciencias de la Salud, Universidad Autónoma de Chile, Temuco, Chile; ^2^ Millennium Institute on Immunology and Immunotherapy. Laboratory of Integrative Biology, Center for Excellence in Translational Medicine-Scientific and Technological Bioresource Nucleus (CEMT-BIOREN), Universidad de La Frontera, Temuco, Chile; ^3^ Departamento de Anatomía Patológica, Universidad de La Frontera, Temuco, Chile

**Keywords:** cytokines, epigenetic regulation, gastric cancer (GC), chemoresistance, angiogenesis, metastasis

## Abstract

Cytokines are proteins that act in the immune response and inflammation and have been associated with the development of some types of cancer, such as gastric cancer (GC). GC is a malignant neoplasm that ranks fifth in incidence and third in cancer-related mortality worldwide, making it a major public health issue. Recent studies have focused on the role these cytokines may play in GC associated with angiogenesis, metastasis, and chemoresistance, which are key factors that can affect carcinogenesis and tumor progression, quality, and patient survival. These inflammatory mediators can be regulated by epigenetic modifications such as DNA methylation, histone protein modification, and non-coding RNA, which results in the silencing or overexpression of key genes in GC, presenting different targets of action, either direct or mediated by modifications in key genes of cytokine-related signaling pathways. This review seeks insight into the relationship between cytokine-associated epigenetic regulation and its potential effects on the different stages of development and chemoresistance in GC.

## 1 Introduction

Gastric cancer (GC) is one of the most frequent solid tumors of the digestive system, with an unfavorable prognosis and a low 5-year survival rate (1). The GC carcinogenesis process has been observed in early stages, such as the development of chronic gastritis and gastric atrophy, which are closely related to the participation of inflammatory mediators and can evolve, causing dysplasia and other morphological modifications that ultimately promote the generation of the tumor (2). Pro-inflammatory and anti-inflammatory cytokines are closely related (3) and act by generating effects on various cell types, regulating processes such as cell death, proliferation, differentiation, and migration (4).

Epigenetic factors may regulate gene expression with its consequent translation and function in cytokines. In epigenetics, the states of active or silent genes are controlled by adding or eliminating chemical modifications in chromatin (3), including DNA methylations (3) and modification of histone proteins, such as acetylation. There are also non-coding RNA (ncRNA), which are RNA that are not translated into proteins, among which are found mainly microRNA (miR) (5) and long non-coding RNA (lncRNA), which play an important role in transcriptional and post-transcriptional regulation, as they target and affect multiple cell signaling pathways by contributing to the development and progression of inflammatory diseases and cancer (5).

The following review will analyze the background linked to the association among the epigenetic regulation of cytokines that affect angiogenesis, progression, metastasis, and chemoresistance of GC.

## 2 Gastric cancer

According to GLOBOCAN 2020, GC is responsible for more than one million new cases and an estimated 769,000 deaths, ranking fifth in incidence and third in mortality globally (6). Associated risk factors include diet, excessive alcohol consumption, socioeconomic status, and infection with the bacterium *Helicobacter pylori* (*H. pylori*) (7). Infection with this bacterium is one of the most studied factors, as it induces chronic inflammation and cell proliferation by increasing the production of chemokines such as CCL5 and expressing pro-inflammatory cytokine genes IL (Interleukin)1, 6, 8, and tumor necrosis factor-alpha (TNF-α), increasing the risk of DNA damage and tumorigenesis (8). Approximately 10% of GC cases are associated with Epstein-Barr virus (EBV), contributing to tumorigenesis through a variety of mechanisms, including hypermethylation of tumor suppressor genes, inflammatory changes in the gastric mucosa, host immune evasion by EBV, and changes in cell cycle pathways (9, 10).

## 3 GC and inflammation

Cytokines are regulatory proteins of immune and inflammatory response (11). They can be produced by leukocytes, fibroblasts, and tumor cells (12), playing a role in cancer development with interferons (IFN), interleukins (IL), colony-stimulating factors (CSF), tumor necrosis factors (TNF), transforming growth factors (TGF), and a wide range of chemokines (13), controlling different stages of cancer, such as apoptosis, angiogenesis, proliferation, invasion, metastasis, and currently the development of chemoresistance (3).

GC cells and the tumor microenvironment contains pro- and anti-inflammatory cytokines that influence tumor growth and the host’s antitumor response (14). Their signaling occurs via a pathway network (14), including Janus-activated kinases (JAK) and activators of transcription (STAT) such as STAT3 (4). Examples such as C-C Motif Chemokine Ligand 5 (CCL5) secreted by type 2 tumor-associated macrophages (TAM) activate STAT3 signaling and DNA methyltransferase (DNMT) by inhibiting gelsolin (GSN) expression, promoting proliferation and the formation of invasion/metastasis (8). Other pathways involved are mitogen-activated protein kinases (MAPK) and nuclear factor kappa B (NF-κB) (4), directly or indirectly affecting epigenetic regulations.

In the tumor microenvironment (TME) context, which consists of neoplastic, mesenchymal, endothelial, immune, extracellular matrix, and fibroblast cells contributing to tumor progression (15) cytokines are relevant. Heterogeneous and functionally reprogrammable TAM in the TME correlate positively with poor prognosis in several cancers (16, 17). Their role in tumor progression is complex (18) and favor angiogenesis and cancer progression by secreting cytokines, growth factors, and proteolytic enzymes (17). Their contribution to drug resistance and post-chemotherapy relapse is important as they suppress cytotoxic T cell immunity, activate anti-apoptotic programs, and polarize macrophages to pro-tumor phenotype (19).

**Figure 1:**
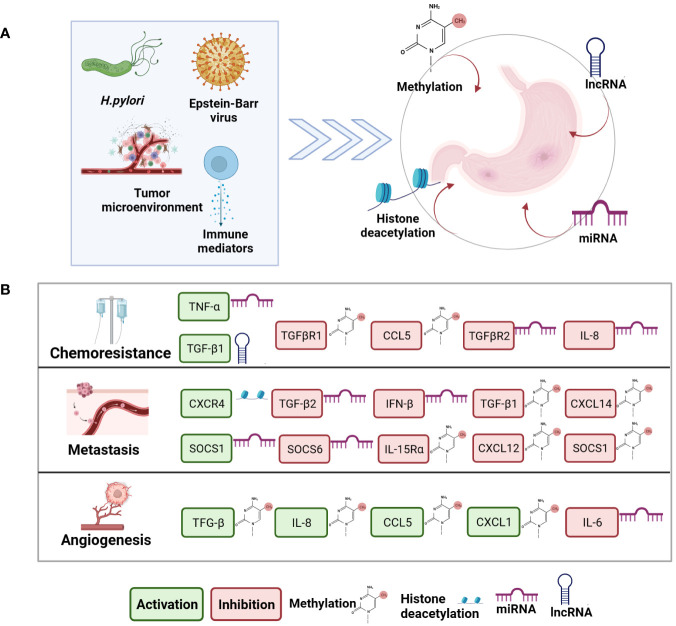
Aberrant epigenetics and epigenetic modulation of cytokines are involved in chemoresistance, angiogenesis, and metastasis in Gastric Cancer. Panel **(A)** Factors that cause abnormal epigenetics in GC. General determinants of epigenetic modifications in cancer, such as infectious agents and components of the tumor microenvironment. **(B)** Epigenetic modulation of cytokine expression in chemoresistance, angiogenesis, and metastasis in GC. Epigenetic modulation includes methylation, histone deacetylation, miRNA, and lncRNA. Green rectangles: cytokine activation. Red rectangles: cytokine inhibition.

Macrophages are classified into M1 and M2 with pro- and anti-inflammatory functions, respectively. In GC, M1 secrete CXCL9 and CXCL10, IL-1β, TNF-α, and IL-8, among others, stimulating tumor growth, while M2 secrete anti-inflammatory cytokines such as IL-33, IL-10, and TGF-β (17). Regulated by epigenetic factors, these cytokines play critical roles in different stages of cancer (**Figure 1**).

It is important to highlight additional functions of these cytokines, besides their role as modulators of inflammation. For instance, they could serve as predictive biomarkers for GC by assessing circulating levels of IL4-IL6 (20, 21). These cytokines are also considered therapeutic targets in GC treatments, influencing the generation of neoplastic transformation and metastasis, and are associated with an increase in TNF-α (22). Moreover, they regulate the secretome, proliferation, and differentiation of epithelial cells (4).

As mentioned above, *H. pylori* is one of the main risk factors in the carcinogenesis of GC, increasing inflammatory processes. *H.pylori* causes an inflammatory reaction in gastric epithelial cells, generating neoplastic deformations and changes in the secretome. In immune cells at the site of infection, the expression of proinflammatory cytokines and the chemokine CCL5 increases, key in the development of chronic gastric inflammation and the onset of GC (23). In a meta-analysis study of the literature, a significant increase in circulating levels of IL-6 and TNF-a was found during infection with the bacteria, when it was also associated with GC, increased serum levels of IL-6 IL-7, IL-10, IL-12 and TNF-a were found (24). Another study demonstrated that the presence of the bacteria favors the activation of nuclear factor kappa B (NF-kB) in gastric epithelial cells, generating the release of inflammatory mediators such as interleukin-8 (IL-8) (25).

## 4 Epigenetic modulations of cytokines in GC

Epigenetics is the study of heritable changes in gene expression not influenced by modification of the primary DNA sequence. Epigenetic mechanisms play a crucial role in the typical development and tissue gene expression in humans. They also govern how an individual’s genotype reacts to and engages with its surroundings. Epigenetic dysregulation, induced by factors like age, cigarette smoking, or the onset of chronic inflammation, may lead to modifications in gene expression and the initiation of cancer development (26). The principal epigenetic mechanism associated with most cancers are:

A) **DNA methylation** by adding methyl groups to the 5’ carbon at the cytosine nitrogenous base binding sites on CpG dinucleotides by DNA methyltransferase (DNMT) enzymes. The inclusion of the methyl group affects gene expression, as the modification prevents specific transcription factors and other transcriptional regulatory components from accessing the promoter region of a particular gene, which suppresses DNA expression (27).
B) **Modification of histones**, mainly acetylation, although they can also be phosphorylated or methylated. Histones are proteins that give structure to DNA, forming chromatin. The DNA coils around these proteins allowing a compact form due to the phosphates in the DNA (negative charge) and that the proteins have a positive charge (abundant amino acid lysine). For gene expression to occur, DNA needs to unwind, and this happens when histones are modified. The acetylation is regulated by histone acetyltransferases (HATs) enzymes that add acetyl to the histone at the lysine position and by neutralizing its positive charge, the DNA is released from packaging and transcription increases. Histone deacetylases (HDACs) remove the lysine acetyl group and maintain the positively charged histones resulting in stability of the coiled chromatin, suppressing gene transcription (28).
C) **Non-coding RNAs (ncRNAs)**, transcribed without translation into proteins. In the context of cancer, microRNAs (miRs) stand out, classified as short ncRNAs of 22-25 nucleotides. They can bind to mRNA through base complementarity, influencing its degradation or translational inhibition, thus regulating post-transcriptional gene expression. On the other hand, there are long non-coding RNAs (lncRNAs) with more than 200 nucleotides in length, which exert multiple gene regulations by targeting mRNA, DNA, proteins, and miRNAs, influencing processes such as transcription, translation, and other epigenetic components (29).

Each of these processes can lead to gene under- or overexpression.

### 4.1 Abnormal epigenetic in GC

The combination of genetic, epigenetic, and environmental elements participate in the carcinogenesis and development of GC. The epigenetic alterations in GC is influenced by various factors such as infection with *H pylori*, EBV, tumor microenvironment and cytokines (30) (**Figure 1A**).

Infection with *H. pylori* causes a prolonged inflammatory reaction of the immune response, *H. pylori* is not only one of the main risk factors influencing the development of inflammation in GC but can also cause epigenetic deregulations, such as histone protein deacetylation (31) and phosphorylation (32). Although its primary impact is on DNA methylation, affecting tumor suppressor genes related to autophagy, whose modification silences them and favors GC (25).

Additionally, *H. pylori* modifies the methylation of genes associated with signaling pathways activated by G protein-coupled receptors, such as guanine nucleotide-binding protein subunit beta-4 (33). Treatments that eradicate the bacteria show a reversal in methylation in patients without intestinal metaplasia (34) In GC, EBV-induced host genome hypermethylation directly targets key tumor suppressor genes, including APC, PTEN, and p14ARF, among others. EBV-induced hypermethylation silences genes that regulate the cell cycle and cell differentiation, leading to increased proliferation and dedifferentiation (35).

The environment around a tumor, known as the tumor microenvironment (TME), is composed of different cell types such as immune cells, fibroblasts, and endothelial cells. It is a key player in tumor formation, tumor growth, and metastasis. Additionally, it influences the treatment response (36).

It has been described that cytokines may be aberrantly regulated by different epigenetic mechanisms in tumor tissues, contributing to carcinogenesis in multiple ways. Some of these cytokines also function as regulators of other genes crucial to tumor biology, so a direct or indirect relationship can occur (3) (37). (**Figure 1B**, **Table 1**).

**Table 1:** Summary of epigenetic modification of cytokine in Gastric Cancer (GC) characteristics.

Gastric Cancer Characteristics	Epigenetic modification	Gastric cancer model	Cytokine involved	Reference
**Angiogenesis**	RNA modifications N6-methyladenosine	GC patient’s dataset	TGF-β	(37)
	Histone lysine demethylase 4	MKN45 GC cell line infected with *H.pylori*	IL-8/CXCL1/CXCL5	(38)
	Dysregulation of miR-149	Cancer-associated fibroblasts (CAFs) of GC, TME	IL-6	(39)
	Overexpression of miR-204	GC patients dataset *H.pylori* – and +	IL-6/IL-8	(40)
**Progression and metastasis**	DNA hypermethylation	GC cell lines MGC-803, BGC-823, SGC-7901. Primary GC tissues	CXCL12	(41)
	DNA hypermethylation	GC cell lines AGS, SGC7901, BGC823, MGC803. GC patients’ tissues	CXCL14	(42)
	DNA hypermethylation	GC cell lines AGS EBV- and + Tissues from GC patients	IL-15Rα	(43)
	DNA Methylation	Patients with early GC and patients with gastritis without GC	SOCS3	(44)
	DNA hypermethylation	GC cell lines AGS, SNU-16, KATO III, MKN28 and MKN45. Primary GC patient’s samples and adjacent non-cancer tissues	IL-6/SOCS1	(45)
	DNA hypermethylation	GC patients’ gastrectomy and non-GC subjects. *H. pylori* infection tested	TGF-β1/IL-1β	(46)
	DNA hypermethylation	GC patients with upfront gastrectomy	SOCS1	(47)
	Histone deacetylation	GC cell lines SGC-7901, MKN-45, AGS and tumor samples from patients	CXCR4/CXCL12	(48)
	Inhibition of miR-139	Gastric cancer cell lines (SGC-7901, MKN-45, AGS) and tumor samples from patients	CXCR4/CXCL12	(48)
	Overexpression of miR-17-5p	GC Cell lines SGC7901 and MKN28. Patients’ samples from gastrectomy	SOCS6	(49)
	Overexpression of miR-370	Patients GC tumor tissues and blood	TGF-β/TGF-βRII	(50)
	Dysregulation of miR-BA5-5p	GC cell lines SNU601, SNU484, SNU216, and SNU719	IFN‐ß	(51)
	Overexpression miR-922	GC cell lines SGC7901, MGC803, MKN45, and HGC-27. Paracarcinoma tissue (>5 cm from the lesion), GC tissue	SOCS1	(52)
**Chemoresistance**	Overexpression miR-135b-5p	GC cell lines MKN45, SNU1 and SNU601. Gastric tissue normal and GC samples	TNFα (NF-κB)	(53)
	Overexpression miR-204	GC cell lines AGS, SGC-7901, MKN-45, MGC-803, and BGC-823. GC tissue and their adjacent non-tumor mucosa from patients	TGF-βRII	(54)
	Hypomethylating agent (5-aza-CdR)	GC cell lines OCUM-2M and MKN-74	TGF-βRI	(55)
	Supresión de DNMT1	GC cell lines AGS, NCI-N87, AZ521, HR, MKN45, and NUGC3. Normal gastric epithelial cell line GES-1. Monocyte lymphoma cell line U937	CCL5/CCR5	(8)
	LncARN MACC1-AS1	GC cell lines (AGS, BGC803, BGC823, MKN45, SGC7901) GC tissue samples from patients.	TGF-β1	(56)

### 4.2 Epigenetics and cytokine regulation in the development of angiogenesis in GC

Angiogenesis (AG) (18) is a process by which new blood vessels are formed, where endothelial cells form tubular structures that bind together to form stable blood vessels (57), which is critical to tumor growth, invasion, and metastasis. GC tumor and stromal cells produce various pro-angiogenic growth factors, such as vascular endothelial and platelet-derived endothelial cells, as well as IL-8 and angiopoietin (8, 18) (**Figure 1B**, **Table 1**).

Epigenetic regulation, like DNA hypermethylation in gene promoter regions, is generally associated with transcriptional silencing, whereas hypomethylation facilitates gene expression (3). Post-transcriptional modifications of mRNA, including N6-**methyl**adenosine (m6A) and 5-**methyl**cytosine (m5C), are involved in mRNA stability, translation, and translocation (58). Wang et al. report that methyltransferase 3-mediated m6A promotes the activation and maturation of dendritic cells involved in the immune response (58). This methylation mechanism is also associated with the activation of several biological pathways, such as transforming growth factor beta (TGF-β) signaling, epithelial-mesenchymal transition (EMT), and chemokine signaling (37).

TGF-β is a cytokine that has a dual function; when the tumor is in early stages it acts as a proliferation suppressor, arresting the cell cycle and promoting apoptosis. In advanced stages of the tumor, it favors the epithelial-mesenchymal transition (EMT). This last process favors the change in cellular characteristics such as intercellular adhesion molecules, which favors invasion and metastasis to other tissues. TGF-β acts through its membrane receptors, allowing its phosphorylation and downstream activation of the SMAD 2/3/4 system that targets the nucleus to regulate proliferation, immune mediators, EMT and metastasis. This pathway becomes central in the interrelation between epigenetic modifications and cytokines in GC (59) and will be developed in subsequent sections.

Another cross-signaling pathway for the interrelation of epigenetics and cytokines in GC is Chemokine receptor signaling. Chemokines are part of the cytokine family, but they are characterized by their function in promoting chemotaxis, that is, they allow the trafficking of leukocytes to be directed towards the tumor and its microenvironment. Chemokines activate receptors found in membranes and are coupled to G proteins, which allows the activation of downstream signaling of MAPK and PI3K in tumor cells, which favors cell survival and proliferation. Additionally, through a pathway independent of Protein G, chemokines activate the JAK/STAT signaling pathway. This pathway is relevant since it allows the regulation of transcription of genes related to inflammation, allowing the cell to regulate itself through autocrine signaling and promote their survival and resistance to drugs (60).

In this sense, activation of EMT and TGF-β in GC leads to decreased T cell trafficking in tumors, modulation of angiogenesis, and activation of fibroblasts (61). In patients with GC positive for *H. pylori*, efficient transcriptional activation of IL-8, CXCL1, and CCL5 has been observed in a histone protein demethylase-dependent manner. Hence, it is implicated as an inducible epigenetic factor under stress conditions and as a contributor to the dysregulation of gastric TME and angiogenesis (38).

In addition, dysregulation of miRNA, such as miR-149 and miR-204, also contributes to angiogenesis in GC. Hypermethylation of the miR-149 promoter region in cancer-associated fibroblasts (CAF) is related to *H. pylori* infection and the activation of Cyclooxygenase-2/Prostaglandin E2 (COX-2/PGE2) signaling, regulating chronic inflammation. PGE2 binds to prostaglandin E receptors and activates downstream signaling pathways such as the β-catenin pathway, the PI3K/AKT pathway, and the NF-κB pathway, which may promote proliferation, survival, and regulation of the immune system. The COX-2/PGE2 pathway favors immune evasion of the tumor, which stimulate not only cell survival, but also chemoresistance (62), resulting in an increased secretion of IL-6 (39). Conversely, miR-204, when overexpressed, inhibits NF-κB pathway-associated genes like IL-6 and IL-8 (40). NF-kB is a transcription-factor involved in cellular immunity, inflammation, and stress. inflammatory responses, is one of the most important molecules linking chronic inflammation with cancer, and its activity is tightly regulated by several mechanisms. NF-κB activation induces several target genes, such as proproliferative and antiapoptotic genes, and NF-κB signaling crosstalk affects many signaling pathways, including those involving STAT3, AP1, interferon regulatory factors, NRF2, Notch, WNT–β-catenin and p53 (63, 64).

Angiogenesis in GC is influenced by epigenetic mechanisms, such as DNA methylation, mRNA post-transcriptional modifications, and miR regulation, underscoring the complexity of the molecular events involved in this process.

### 4.3 Epigenetics and cytokine regulation in GC progression and metastasis

GC progression and metastasis are critical aspects that arise mainly due to the late diagnosis of the disease. The potential of genetically unstable tumor cells to invade adjacent tissues and migrate to distant organs is known as metastasis (65). This process is associated with altered cell adhesion, cell motility, invasion, resistance to cell death signals, basement membrane disruption, and extracellular matrix (65).

In the context of epigenetics and cytokine regulation in GC progression and metastasis, it has been noted that epigenetic silencing can influence the loss and imbalance in expression levels of the chemokine CXCL12 and its receptor, CXCR4. Hypermethylation of the CXCL12 promoter has been associated with inhibition of tumor metastasis, and restoration of expression by methyltransferase inhibitors has been shown to reverse this effect (41, 66). Using the same methodology, Hu et al. proved that the chemokine CXCL14 could be involved in the development and progression of GC. CXCL14 was reduced in GC tissues compared to normal tissues. Abnormal hypermethylation of the promoter region in tumor tissue is one of the mechanisms causing the reduction. Promoter demethylation has been shown to restore CXCL14 expression, correlating positively with the prognosis in stages III/IV (42).

TGF- β1 (anti-inflammatory cytokine) has biphasic effects on tumorigenesis (67), acting as a tumor suppressor in the early stages and promoting tumor progression in the late stages. Methylation of the TGF-β1 promoter has been linked to the development of various solid tumors, including GC. Wang et al. showed that high methylation levels in patients with GC who test positive for *H. pylori* are associated with the increase and production of proinflammatory cytokines such as IL-1β (46).

Expression of suppressor of cytokine signaling-1 (SOCS1) prevents the activation of the JAK/STAT signaling pathway (a pathway that promotes tumor development) (68). Loss of SOCS1 expression through promoter hypermethylation is strongly associated with the overproduction of inflammatory cytokines such as TNF-α and IL-6 (69). This loss of expression contributes to the activation of the JAK/STAT pathway and tumor progression; its demethylation restores SOCS1 expression in GC and suppresses constitutive STAT3 phosphorylation (45), like what occurs with SOCS3 (44). In addition, studies have shown that downregulation of SOCS1 by promoter hypermethylation is related to infection by *H. pylori* and the generation of inflammatory cytokines during gastric carcinogenesis (47).

In the case of EBV-positive GC, cells expressed the interleukin IL-15Rα receptor binds to IL 15 and allows the increase of natural killer cells, in addition to the regulation of the JAK/STAT pathway and thyrosine kinase pathways such as MAPK and PI3K (70), at a lower level than EBV-negative cells due to promoter hypermethylation (43); therefore, administration of IL-15 to stimulate immune responses against cancer could be a promising strategy in GC.

In the context of epigenetic mechanisms associated with miRNA in GC progression and metastasis, an interaction has been found between human epidermal growth factor receptor 2 (HER2) is a central tyrosine kinase receptor in cancer development, has epidermal growth factor (EGF) among its ligands and allows activation of a series of downstream signaling pathways such as MAPK, PI3K and PKC, influencing at the level of nuclear transcription and modulating cell-cycle progression, proliferation, and survival (71) and CD44 that contributes to metastasis by deacetylating histones, which suppresses the transcription of miR-139. This miR represses the chemokine receptor CXCR4, thereby decreasing tumor invasion and growth (48). Treatment with trastuzumab, a drug that inhibits HER2, has been shown to restore CXCR4-associated miR-139 and reduce invasiveness (48).

Other miRs, such as miR-17-5p (49) and miR-370 (50), have also been implicated in GC progression and metastasis by regulating genes such as SOCS6 and TGF-β-RII, respectively. Reduced patient survival has been linked to overexpression of miR-370, indicating the potential significance of this biomarker (50).

miR-922, which has been found to be overexpressed in GC, targets the SOCS1 gene and negatively regulates its expression by activating the JAK and AKT pathways, promoting tumor cell proliferation and motility. Conversely, downregulation of miR-922 increases SOCS1 expression and promotes the apoptosis of GC cells (52).

GC-associated EBV-encoded miRs, such as miR-BART6-3p, have also been linked to the carcinogenesis and progression of malignant neoplasms by suppressing IFN-β production and targeting genes like retinoic acid-inducible gene I (*RIG1)* (51). This activates a signaling cascade downstream and leads to the production of type I interferons, proinflammatory cytokines (72), and IL-6R (73), causing the immune system to deteriorate.

### 4.4 Epigenetics and cytokine regulation in GC chemoresistance

Chemoresistance in GC can arise from inherent mechanisms inside the tumor cell or can be acquired during treatment. Classic mechanisms include abnormalities in cell membrane transporters, increased DNA repair, reduced apoptosis, presence of tumor stem cells, changes in detoxification enzymes, disorders in miR regulation, development of EMT, and hypoxic conditions (74). Components of the tumor microenvironment and cytokine secretion also generate chemoresistance (75) (**Table 1**).

TGF-β acts as a tumor suppressor, but tumor cells can develop resistance to its inhibitory effects. Hypermethylation of the TGF-βRI receptor is associated with resistance to TGF-β function in GC (76). Demethylation can increase TGF-βRI expression, suggesting a synergistic role with anticancer drugs. TGF-β is associated with sensitivity to chemotherapy (55).

Tumor-associated macrophage (TAM) infiltration in GC tissues is associated with high expression of DNMT1 (77). M2 macrophages upregulate DNMT1, and suppression of DNMT1 has antitumor effects related to the inhibition of CCR5 involvement stimulated by CCL5 (8). DNMT1 inhibition with 5-AZA or the C-C chemokine receptor (CCR) antagonist (Maraviroc) could be a therapy option with anti-inflammatory effect.

In resistance to cisplatin in GC, an *H. pylori* infection produces high levels of inflammatory cytokines such as TNF-α (78) and induces miR-135b-5p production by suppressing KLF4 (Kruppel-like factor 4) is a transcription factor that regulates proliferation, apoptosis, inflammation, and tumorigenesis, acting as a tumor suppressor in gastrointestinal tumors (79). The repression of which decreases apoptosis and increases drug resistance (53). In contrast, miR-204 acts as a tumor suppressor, sensitizing GC cells to 5-FU by suppressing the TGF-β-mediated EMT signaling pathway (54).

lncRNA also play a role in chemoresistance in GC. The overexpression of the lncRNA MACC1-AS1 in GC tissues is caused by TGF-β secreted by mesenchymal stem cells (MSC) through immune response-associated SMAD 2/3 activation, which promotes proliferation and chemoresistance by inhibiting miR-145-5p (56).

## 5 Epigenetic/immune target therapy in GC

The main epigenetic modifications in CG that regulate cytokines involve hypermethylation, which can lead to the repression of the function of modulated genes. One of the primary executors of these modifications is the DNA methyltransferase (DNMT) enzymes, making them the targets in this therapy (80). Methylation is a reversible process, and DNA methylation inhibitors were examined as anti-tumoral agents to demethylate and reverse suppressed genes. 5-azacytidine (azacytidine) (81) and 5-aza-2’-deoxycytidine (Decitabine) (80) are two cytosine analogs acting as DNMT inhibitors, approved by the Food and Drug Administration (FDA) as anti-tumor drugs in 2004 and 2006, respectively.

For GC, 5-azacitidine, has been used in administration as a single therapy in preclinical trials with an *in vivo* gerbil model, showing a decrease in global hypermethylation and the incidence of GC (82). In the case of clinical trials, it has been administered as a combination therapy of Azacytidine with capecitabine/epirubicin/oxaliplatin (83).

In relation to histone modification, the therapy targets are deacetylase enzymes. Deacetylase enzymes remove acetyl groups, increasing the positive charge of histones and their affinity for DNA. This increased binding condenses the chromatin structure, preventing gene transcription. The epigenetic therapy is based on using deacetylase enzyme inhibitors with drugs such as Vorinostat (84), which is approved by the FDA.

For GC, valproic acid has been used in administration as a single therapy in preclinical trials with *in vivo* model of xenograft tumor in BALB/c nude mice showing suppression of cellular proliferation and initiation of programmed cell death (85). On the other hand, clinical trials have been conducted using combination therapy with Vorinostat in conjunction with cisplatin and capecitabine (86).

In the case of miRNA, the main strategies for targeting miRNAs are oligonucleotides inhibiting miRNA, miRNA sponges and inhibitors in the form of small molecules. miRNAs can exhibit therapeutic efficacy either alone or in combination with any drug. Combination therapy has been documented to target a broader range of tumors, induce therapeutic efficacy and overcome drug resistance (87).

The merging of immunotherapy and epigenetic medications has gained prominence in cancer treatment research in recent years, with the most notable being the fusion of immune checkpoint blockade therapy and epigenetics. For instance, azacytidine demonstrated the ability to increase the expression of the PD-L1 gene both at the transcriptional level and directly on the cell surface in an *in vitro* model of lung cancer cells. Recognizing the application of epigenetic therapies in checkpoint inhibitor therapy could enhance immune responses more effectively (88).

## 6 Conclusion

When establishing the link between epigenetic regulation and inflammation or immune response, the importance of the epigenetic target as a crucial player in therapy is emphasized. Controlling this factor enables the direct or indirect regulation of the immune response. Epigenetic alterations, especially DNA hypermethylation, affect key genes and pathways associated with immune response, influencing angiogenesis essential for tumor growth and metastasis in GC progression. In GC progression and metastasis, epigenetic silencing influence, for example, the expression of chemokines like CXCL12 and its receptor, CXCR4. Examining epigenetic mechanisms in GC chemoresistance reveals the involvement of TGF-β, tumor-associated macrophages, and specific miRNAs in influencing chemotherapy response. The comprehension of the epigenetic and cytokine landscape in GC provides valuable insights for developing targeted therapies. The use of DNA methyltransferase and histone deacetylase inhibitors emerges as a promising therapeutic approach in GC.

## Author contributions

MR: Conceptualization, Formal analysis, Visualization, Writing – original draft. VP: Data curation, Investigation, Writing – original draft. CV: Formal analysis, Investigation, Writing – review & editing. CI: Formal analysis, Validation, Writing – review & editing. BM-L: Data curation, Funding acquisition, Writing – review & editing. PB: Funding acquisition, Writing – review & editing.
